# Local and scientific knowledge for assessing the use of fallows and mature forest by large mammals in SE Brazil: identifying singularities in folkecology

**DOI:** 10.1186/1746-4269-10-7

**Published:** 2014-01-10

**Authors:** Helbert Medeiros Prado, Rui Sérgio Sereni Murrieta, Cristina Adams, Eduardo Sonnewend Brondizio

**Affiliations:** 1 Laboratory of Human Evolutionary Studies, Department of Ecology, Biosciences Institute, University of São Paulo, 277 Matão Str., São Paulo, SP 05508-090, Brazil; 2 Laboratory of Human Evolutionary Studies, Department of Genetics and Evolutionary Biology, Biosciences Institute, University of São Paulo, São Paulo, Brazil; 3 Laboratory of Human Ecology and Center for Interdisciplinary Research on Complex Systems (NISC-USP), University of São Paulo, 1000 Arlindo Bétio Ave., São Paulo, SP 03828-000, Brazil; 4 Department of Anthropology, Anthropological Center for Training and Research on Global Environmental Change, Indiana University, 701 E. Kirkwood Ave., Bloomington IN 47405-7100, USA

**Keywords:** Ethnoecology, Local ecological knowledge, *Quilombola* populations, Shifting cultivation, Medium and large-bodied mammals, Camera trap, Secondary forest, Brazilian atlantic forest

## Abstract

**Background:**

Local ecological knowledge (LEK) has been discussed in terms of its similarities to and its potential to complement normative scientific knowledge. In this study, we compared the knowledge of a Brazilian *quilombola* population regarding the habitat use and life habits of large mammals with *in situ* recordings of the species. We also tested the hypothesis that *quilombola* LEK has a special focus on the anthropogenic portion of the landscape.

**Methods:**

The habitats investigated were anthropogenic secondary forests and mature forests in the southeastern Atlantic coast of Brazil. We conducted the faunal survey using the camera-trap method. The sampling effort consisted of deploying 1,217 cameras/day in the mature forests and 1,189 cameras/day in the secondary forests. Statistical comparisons regarding the habitat use of the species were based on the randomization procedure. We interviewed 36 men who were more than 40 years old in the three communities studied. Informal, semi-structured and structured interviews were used. Two variables were considered in the LEK analyses: level of internal agreement and level of convergence with the scientific data.

**Results:**

The camera trap sampling resulted in a total of 981 records. Animals such as opossums, tayras, armadillos and deer showed a non-selective pattern in the use of habitats. In contrast, the coati was more common in mature forests. We found that nearly 40% of the interviewees’ responses converged with the scientific data on the use of habitats. However, the LEK on the species’ life habits was highly convergent with the scientific data. The hypothesis that secondary forests would have a greater relevance for local knowledge was validated for four of the five analyzed species.

**Conclusions:**

We suggest two principal considerations of ecological and ethnoecological interest: (1) In the Atlantic Forest of the Ribeira Valley, the secondary forests resulting from shifting cultivation were as attractive to the species as the mature forests; (2) The LEK has a special focus on the more anthropogenic portion of the landscape studied. Finally, we argue that this environmental focus in LEK is part of what makes it different from scientific knowledge and unique in its approach toward local environments.

## Background

A recent study conducted in French Guiana reported that the knowledge of Wayãpi Indians converged with scientific records on the diet of lowland tapirs (*Tapirus terrestris*), with approximately 70% agreement [1]. Indeed, the similarities and potential complementarity between local and scientific knowledge are recognized in various fields of biology and ecology [2-5].

Local ecological knowledge (LEK) may be defined as the knowledge set of a given population on ecological aspects of the environment and the various practical implications of that knowledge [6,7]. LEK may be shared extensively or only partially by the population members [8-10], and in some cases, it is useful for resource conservation and management [11].

Studies that explicitly compare LEK and ecological research on patterns of space use and abundance of vertebrate species have focused primarily on fish [12-16] and birds [17-19]. Among land mammals, only the caribou (*Rangifer tarandus*) [20] and the artic fox (*Vulpes lagopus*) [19] have been the focus of such studies.

Studies on this subject show that LEK provides data with greater temporal depth, involving fluctuations in species abundance, and it tends to recognize a greater variety of habitats used by the animals [12,15,18-22]. In contrast, ecological studies can complement LEK by assessing regional data on the occurrence of the species [13-17,19,20,23].

The convergences and divergences between LEK and scientific knowledge can be assessed when these knowledge systems are compared on the same observational scale (or regarding the same habitats) [19,24]. This approach also enables LEK to be characterized in terms of its uniqueness in relation to scientific observations.

In the present study, we compared the knowledge of a *quilombola* Brazilian population regarding the habitat use and life habits (diurnal/nocturnal) of 12 large-bodied mammals to *in situ* recordings of local species. More specifically, we tested the hypothesis that the anthropogenic portion of the landscape studied (secondary forests) would have a greater relevance for local knowledge in comparison to mature forests (i.e., we expected secondary forest to be more frequently cited as preferentially used by the fauna than mature forest).

The formulation of this hypothesis is a result of prior studies showing that the history of this *quilombola* occupation was more closely associated with secondary forests than with mature ones in the landscape [25-28], with implications for the LEK about faunal foraging in the area [29]. In fact, until the early 1980s, the secondary forests were the principal context related to the communities’ main subsistence activities, including farming areas, fallows and home gardens.

In addition, by assessing large mammals’ use of mature and fallow forest, we provided additional data to the current debate on the role of secondary forest [30] and, especially, of shifting cultivation [31] in patterns of landscape use by fauna.

### Ribeira Valley and its *quilombola* communities

**Figure 1:**
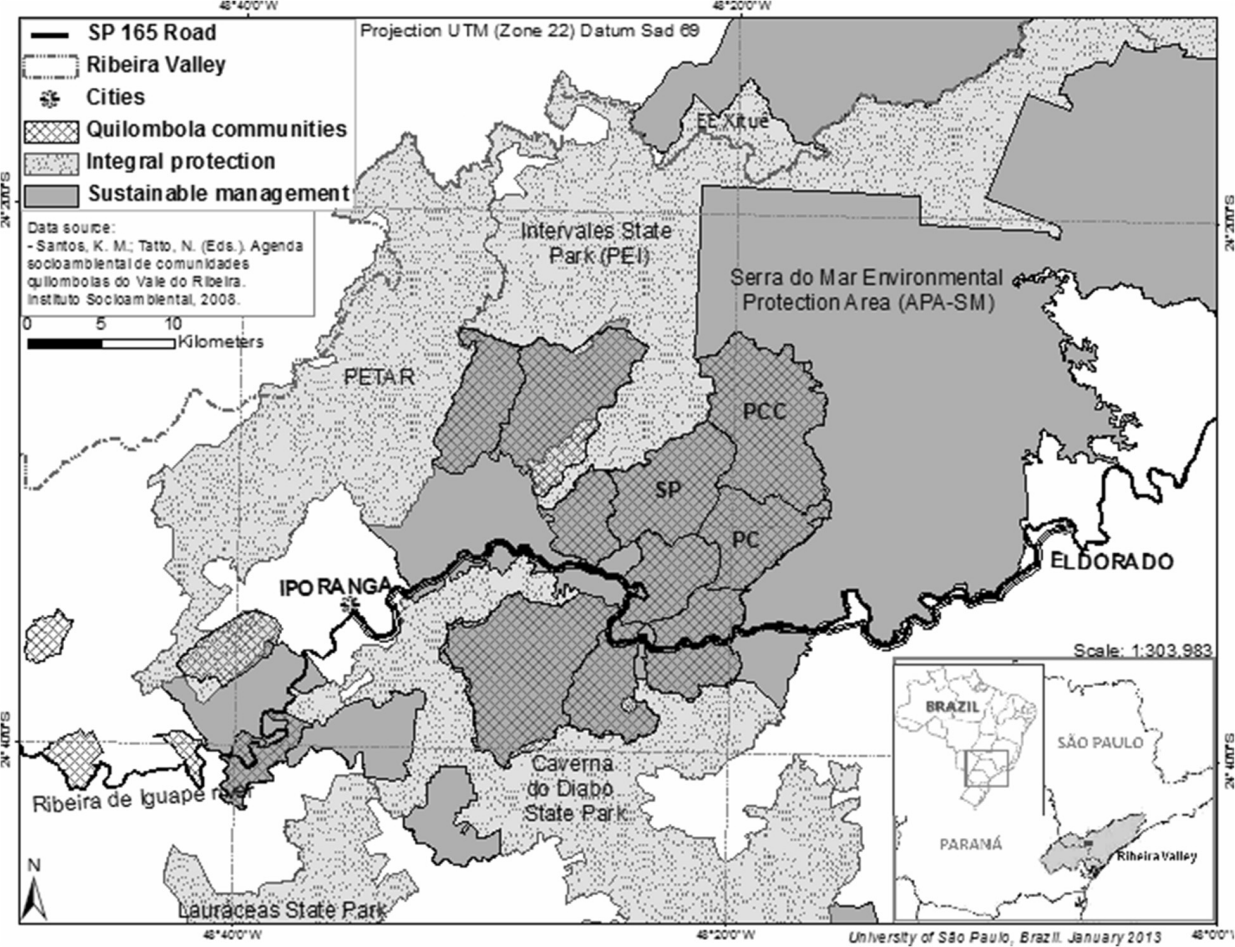
**Studied *quilombola *** **communities and surrounding protected areas (Ribeira Valley, São Paulo, Brazil).**

The Ribeira Valley is located in the southeast São Paulo and northeast Paraná states (Brazil) and has an area of 2,830,666 ha (Figure 1). The vegetation in the valley is Atlantic rainforest, one of the world’s biodiversity hotspots [32], and the region has a tropical monsoon climate (Am Köppen). The annual temperature varies between 17.4°C and 30.4°C (average 23.9°C), and the mean annual rainfall is 1,521.5 mm, concentrated in the summer (January–March) [33].

This region is the economically poorest and most sparsely populated region in São Paulo State [34]. A large portion of the Ribeira Valley is protected by environmental laws and consists of protected areas, forming an ecological mosaic that spans more than 120,000 ha of the Atlantic Forest. The Ribeira Valley is also home to a large number of communities with remaining slave descendants, known as *remanescentes de quilombos* (*quilombo* remmants) (Figure 1). These communities are recognized as local groups whose production systems are based on subsistence agriculture [25,35,36].

The *quilombola* communities of the Ribeira Valley were originally formed by runaway or freed slaves and those who were abandoned during the slave-driven colonial period in Brazil (mid-18th century) [26,36]. In fact, these communities derive historically from a blend of African, European and Amerindian ancestry, and their culture is similar to that of other peasant people in Brazil [36]. Since their formation, shifting cultivation has been their subsistence mainstay, complemented by raising pigs and chickens, hunting and fishing [26,27].

Shifting cultivation is a system in which small areas are cleared with a slash-and-burn technique and then farmed for short periods of time before being left fallow for longer intervals [37]. This practice is found throughout the tropics and in some subtropical regions [38,39]. In the Ribeira Valley, the landscape has been shaped by 200 years of shifting *quilombola* cultivation practices [25].

Nevertheless, as in other places [39], the *quilombola* shifting cultivation system has been changing since the 1960s [27], with impacts on the landscape and livelihoods. In the mid-1960s, household units began to evolve from a scattered landscape occupation (which was based almost exclusively on subsistence agriculture) to a more settled and restricted occupation in the form of hamlets or villages. These transformations resulted in greater interaction between these communities and urban resources and values, to the detriment of past customs and experiences that were more deeply connected with the ecological aspects of the environment (such as subsistence agriculture, raising animals and hunting practices) [25].

The present study was conducted in the São Pedro, Pedro Cubas and Pedro Cubas de Cima communities (Figure 1). São Pedro and Pedro Cubas received official land titles in 2001. Pedro Cubas de Cima has been recognized as a *quilombo* remnant since 2003 but has yet to receive an official land title for the area [25,28]. For additional information about *quilombola* communities in Brazil, see [40] and [41].

The territory officially belonging to the São Pedro community consists of 4,688.28 ha [28]. Less than 1% of this area is occupied by agricultural forms of land use and other features such as bamboo stands and roads, 4% is pasture and roughly 95% is native Atlantic Forest vegetation [28] (data for 2007). This vegetation is highly heterogeneous, with patches of secondary cover in early, mid and advanced stages of succession [28]. Today, the São Pedro community has a population of 120 scattered among 28 households [27].

Pedro Cubas and Pedro Cubas de Cima together occupy 10,681.55 ha, of which slightly more than 3% is devoted to pasture and 4% is devoted to agriculture and other features such as water ways and roads, while 93% supports native vegetation [28] (data for 2007) in various stages of regrowth [28]. The population totals 270 individuals in 68 households [27].

Pre-research consultation visits to the communities were conducted, including meetings with community representatives and community organizations. São Pedro, Pedro Cubas and Pedro Cubas de Cima associations of residents authorized this study, and the Bioscience Institute (University of São Paulo) Ethical Committee approved this research. We have also obtained prior informed consent from each interviewee. Finally, the interviews were scheduled in advance and conducted at the individuals’ residences.

### The studied species

In the present study, we focused on species of medium- and large-bodied terrestrial mammals found in the region, namely the lowland tapir (*Tapirus terrestris*), red and gray brocket deer (*Mazama americana* and *Mazama gouazoubira*), collared peccary (*Pecari tajacu*), white-lipped peccary (*Tayassu pecari*), tayra (*Eira barbara*), ring-tailed coati (*Nasua nasua*), crab-eating raccoon (*Procyon cancrivorus*), lowland paca (*Cuniculus paca*), Azara’s agouti (*Dasyprocta azarae*), crab-eating fox (*Cerdocyon thous*), big-eared opossum (*Didelphis aurita*) and armadillo (*Dasypus sp*.) [42].

In the Neotropics, large mammals are generally among the species that tend to come in contact with human populations most frequently, given their high occurrence within the set of hunted species and their obvious importance in the regional diet [43-46]. For this reason, these species are useful for the comparison between scientific and indigenous ecological knowledge, as exemplified by [1].

## Methods

### Local landscape and study design

Based on maps of the areas inhabited by Ribeira Valley *quilombolas*, aerial photos and workshops conducted with the local population [28], the landscape of the three communities studied herein can be divided into two main general categories. One category, wherein the landscape predominantly comprises forests that are either mature or in advanced stages of forest regeneration [28], is locally known as a *mata virgem* (virgin forest) environment and regarded by locals as areas that have never been deforested for farming [26]. In this article, this landscape context will be called a “continuum of mature forests (MF)”. The other category is the area historically used for farming, where there is a predominance of secondary forests at different stages of regeneration (locally called *capoeiras*). This category will be called here the “mosaic of *capoeiras* (MC).”

We chose to sample at least two types of *capoeiras* that were clearly distinct in terms of vegetation structure, considering the generalist character and high mobility of the mammals discussed here. This strategy was adopted to increase the chances of identifying the species’ habitat-selection patterns when such selection indeed occurred in the landscape. Therefore, we chose two extreme (early-stage and advanced-regeneration *capoeiras*) and one intermediate stage of forest regeneration for sampling fauna.

The identification of these types of environments resulted from the authors’ participation in previous studies of the history of human occupation of the landscape [26] and botanical surveys in secondary forests [47], as well as visits to dozens of *capoeiras* at various stages of re-growth.

These environments were classified by asking informants to identify in the landscape the *capoeiras* with the shortest and longest fallow period. From this procedure, we selected a sample of 10 *capoeiras* with ages ranging from 3 to 7 years of being left fallow. Likewise, we selected 10 additional *capoeiras* ranging between 60 and 70 years of age. Additionally, we selected 10 more *capoeiras* with intermediate ages, ranging from 30 to 40 years of age.

When we chose these *capoeiras*, there was no botanical research on the area to better support our choice. Despite this limitation, at the beginning of our fieldwork, we considered that the intermediate (30–40 years) and advanced (60–70 years) *capoeiras* could be different in terms of vegetation structure. However, this scientific information was provided only at the end of our study [47]. By then, we realized that these two categories were more similar than we had previously thought.

According to [47], in general, we can describe the local *capoeiras* aged between 3 and 7 years as having a high density of individuals (between 4,000 and 7,000 ind/ha), high dominance of few species (primarily small trees and pioneer shrubs of the Asteraceae family), a median basal area ranging between 4 and 12 m^2^/ha, and a maximum median tree height ranging from 3 to 6 m [47].

In addition, [47] characterized the intermediate and advanced-regeneration *capoeiras* as having increased evenness in species abundance patterns, median densities from 3,500 to 4,500 ind/ha, basal areas from 35 to 40 m^2^/ha and median maximum canopy heights from 18 to 20 m. The most abundant plants of that regeneration age category are understory shrubs of the genus *Psychotria* (Rubiaceae) [47]. The vegetation found in the mature forests, the *mata virgem* according to the local designation, was not included in that botanical sample.

### Faunal record

We performed two comparisons regarding the frequency of mammals in different environments: (1) one between the MC and MF contexts and (2) another between the three types of *capoeiras* found in the MC context, initial (C1), medium- (C2) and advanced-regeneration stage (C3) *capoeiras*.

We also conducted a series of informal conversations and hikes with residents (within the previously defined categories) aiming to identify landscape areas suitable for fauna surveys, following a block (or paired) design. Each block referred to a portion of the landscape consisting of an MC patch near the MF context.

**Figure 2:**
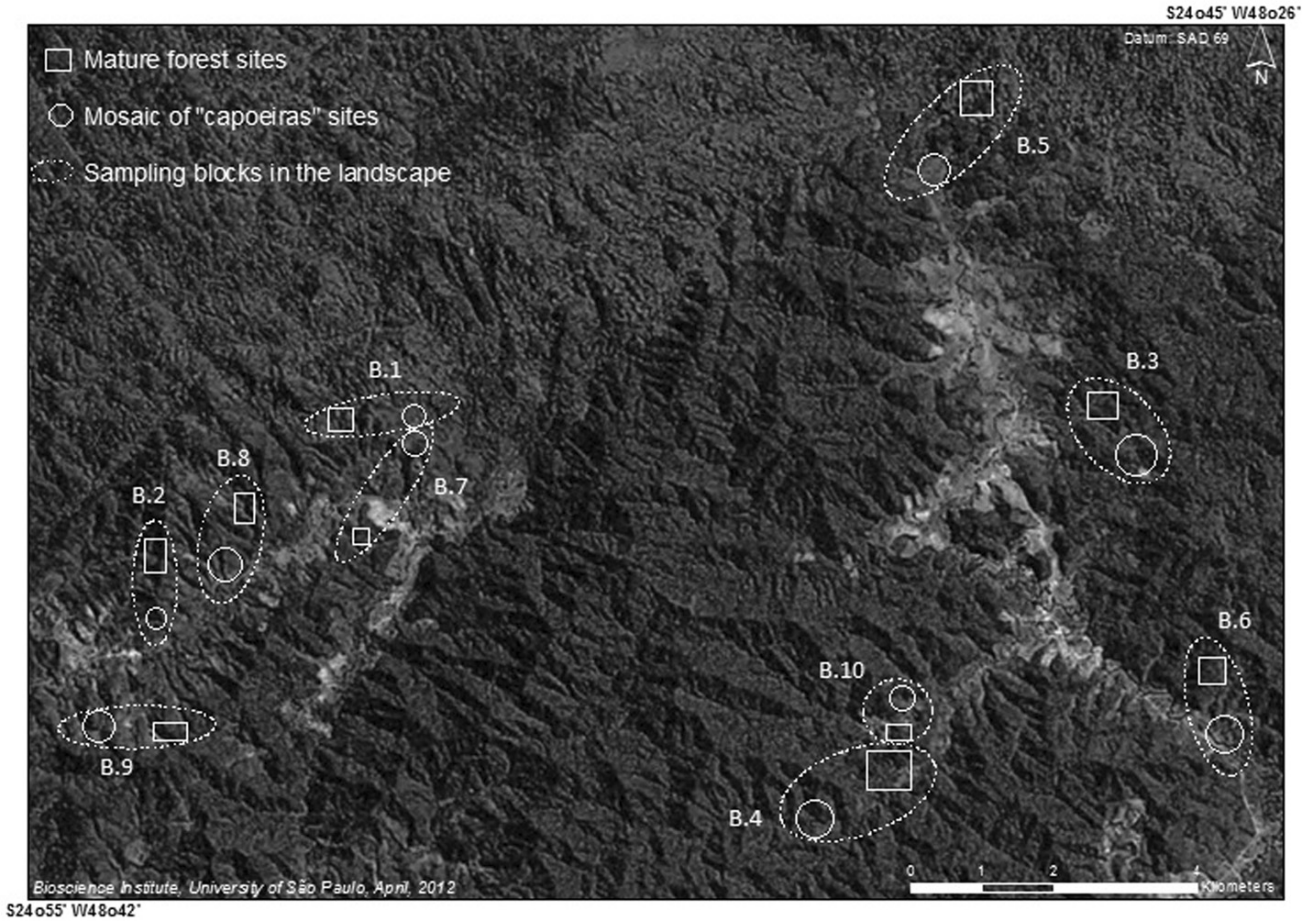
**Distribution of faunal sampling blocks in the landscape (*quilombola *** **areas in the Ribeira Valley).**

This block design facilitated 10 pairwise comparisons between the MC and MF conditions in the area. We chose the blocks according to the following criterion: occurrence of C1, C2 and C3 areas that were between 100 and 200 m apart in the MC context, with portions of the MF continuum that were 500 and 1000 m away from the corresponding MC (Figure 2).

We conducted the faunal survey using the camera-trap method. This method consists of using cameras coupled with motion- and heat-sensitive infrared detectors [48,49]. This technique enabled the *in situ* species surveys to sample the same habitats covered in the interviews by facilitating standardized spatial sampling [49]. The lack of that type of standardization has been indicated as one of the main difficulties in effectively comparing these two sets of knowledge [24].

We positioned a camera in the center of each of the three *capoeiras* sampled in each MC. The distance between the cameras installed in the MC was used for their positioning in the corresponding MF. The species recording comprised two periods. First, we sampled six blocks (B.1 to B.6) between January, 2010 and August, 2011. During this period, we sampled each pair of MF and MC blocks simultaneously during three sessions of 15 days each. Second, we surveyed the fauna in the other four blocks (B.7 to B.10) for 45 continuous days, between August and October, 2011.

The sampling effort consisted of deploying 1,217 cameras/day in the MF context and 1,189 cameras/day in the MC environment. The sampling effort in the type C1, C2 and C3 *capoeiras* resulted in the use of 396, 393 and 400 cameras/day, respectively. We positioned all of the camera traps 15 to 25 cm from the floor. We used banana and coarse salt as bait and reset the traps weekly. We only considered photographic recordings with a minimum interval of one day as independent recordings of the species’ habitat use.

### Ethnoecological data collection

We interviewed 36 people in total: 14 in the community of São Pedro 12 in Pedro Cubas and 10 in Pedro Cubas de Cima, all of whom were men over the age of 40 years. The gender profiling resulted from life-history reports indicating greater involvement of men in the local hunting activities [26,27]. Conversely, the age profiling aimed to record the theoretically more extensive LEK repertoire to compare it with the *in situ* faunal recording in the area. People aged more than 40 years comprise the last generation before the establishment of structural changes in the economic and socio-cultural organization of the communities studied, as previously mentioned [25-27].

Accordingly, we assumed as a premise for conducting this study that the younger generations (up to 40 years old) were educated in a qualitatively different (and more urban) social and cultural context compared to the older ones. The members of the older generations likely gathered a more comprehensive knowledge about their environment, as found in other contexts [50,51], because they had a more intense experience with ecological elements of the landscape [26,27]. The sample of 36 people represents approximately 90% of the total number of individuals who were more than 40 years old in the three communities studied [27]. Of the remaining 10%, four individuals were not interviewed because they were living out of their communities during our research, and one refused to participate in this study.

The age of the interviewees ranged from 40 to 85 years old. We interviewed nine individuals between 40 and 50 years old, nine from 51 to 60, nine from 61 to 70, seven from 71 to 80 and two who were more than 80 years old.

The ethnoecological survey was divided into two phases. First, we surveyed people’s exploratory knowledge about the species of large mammals and their ecological interactions through informal and semi-structured interviews and using data collected in previous studies at the site [25,27].

In the second stage of sampling, we developed a structured closed-ended questionnaire regarding the biological and ecological aspects of the species, including diet, period of activity and habitat use (totaling 10 questions). We considered each species separately in the interviews. On average, each interview lasted approximately 1 hour and 30 minutes. We recorded the data on field sheets and recorders.

In this article, we are considering only data on the habitat use and life habits (diurnal/nocturnal) of the species. The same types of environments that we selected for the *in situ* records of the species were also discussed in the interviews to access the LEK about the habitat-use patterns of the mammals. We conveyed the logic adopted in the faunal survey in the landscape to the interviewees to ensure that they were aware of the level of interest in this research. We informed each resident that the main aim of the interview was to understand how animals move between the mosaic and mature forest environments, which are 500 m to 1 km apart, before addressing the specific questions on habitat use. We used the same type of explanation to address the three categories of *capoeiras* in the questionnaire.

We used two consecutive questions: (1) “Considering the landscape here where we find the environment comprises *capoeiras* close to the *virgin forest* environment, where does the animal appear the most, or does the animal visit those two environments with the same frequency?”; and (2) “When the animal comes to a *capoeira*, do you think it more frequently visits the younger ones, aged approximately from 3 to 7 years, those aged approximately 30 to 40 years or those that are much older, approximately 60 to 70 years, or does it visit those different types of *capoeiras* similarly?” In the scope of the longer questionnaire, we used a question regarding the period of species activity as follows: “Is that animal more active (or searches for food more frequently) during the daytime or nighttime, or is there no difference regarding that activity?”

We used the following codification on field sheets: “1” for affirmative responses and “0” for the negative ones for each environmental category or periods of species activity asked in the interviews. We repeated all the questions for all the interviewees herein considered. Finally, because hunting is illegal in the Brazilian Protected Areas and part of the *quilombola* land falls under this category [25], we did not include this specific topic in the interviews. We found this to be a way of avoiding a sensitive topic and protecting our interviewees from any legal trouble. Thus, we could not address the relationship between individuals’ involvement in hunting practices and their repertories regarding the mammals herein analyzed.

### Analytical procedures

As noted earlier, the two oldest categories of *capoeira* sampled (30–40 and 60–70 years) are very similar in terms of vegetation structure [47]. However, we kept these categories separated in the analysis, which does not affect our main focus on the comparison of the two most extreme ages of *capoeira* (3–7 and 60–70 years). Instead, keeping the intermediate stage as a unique category, we are applying a more detailed analysis, searching for subtle differences in the patterns of landscape use by fauna.

Thus, the independent variables for both ecological and ethnoecological analyses were the environmental categories MM, MC, C1, C2 and C3. The dependent variable involved specifically in the faunal survey was the “number of species recordings”, representing the frequency of use of the various environments by the mammals. The dependent variable of the LEK analyses was the percentage of interviewees who chose each of the environmental categories or periods of species activity mentioned in the questions.

Statistical comparisons regarding the use of MFs and MCs of the species were based on the randomization procedure through 100 re-samplings without replacement, respecting the paired sample design adopted here. The nonparametric Friedman test for paired designs was used for comparisons involving the C1, C2 and C3 types of *capoeira*[52].

We considered two variables in the LEK analyses: (1) the “degree (or level) of internal agreement”, regarding the highest degree of agreement in the interviewees’ answers. This variable was measured in terms of the highest percentage of interviewees who agreed when answering a given question; and (2) the “degree (or level) of convergence”, regarding the highest degree of convergence of the answers with the scientific data (*in situ* recording of species). This variable was measured in terms of the percentage of the interviewees reporting a species’ pattern of use and preference for environments that was also shown in the faunal survey. We organized all data in Excel spreadsheets and performed the analyses using the R software [52].

## Results

The interviewees’ reported frequent encounters with large mammals in *capoeiras*, cultivated fields and old home gardens. When asked how they gathered their LEK, the *quilombolas* mentioned the direct or indirect observation of species’ behavior in the environment and teachings handed down by their elders. Also common were accounts of hunting strategies either for subsistence or in defense of the home gardens and cultivated fields often raided by these species, although these stories are told as incidents that took place in the past.

We gained 981 records of medium- and large-bodied mammals distributed in the MF, MC and other *capoeiras* (Table 1). The opossum and tayra were the most common species in the area. The crab-eating fox, lowland tapir and collared peccary were rare, and there were no recordings of the white-lipped peccary.

**Table 1:** **Number of mammals’ recorded in each environment sampled in *quilombola *** **areas in the Ribeira Valley**

**Species**	**Local name**	**Total**	**MF**	**MC**	**C1**	**C2**	**C3**
*D. aurita*	Opossum	463	239	224	83	74	67
*E. Barbara*	Tayra	274	124	150	44	47	59
*C. paca*	Lowland paca	74	47	27	13	6	8
*N. nasua*	Ring-tailed coati	41	27	14	3	5	6
*D. azarae*	Azara’s agouti	32	0	32	3	7	22
*Mazama sp.*	Brocket deer	30	15	15	8	2	5
*n.i.*	Armadillo	27	7	20	11	4	5
*P. cancrivorus*	Raccoon	23	12	11	8	3	0
*C. thous*	Crab-eating fox	9	0	9	4	5	0
*T. terrestris*	Lowland tapir	5	3	2	2	0	0
*P. tajacu*	C. peccary	3	0	3	0	1	2
*T. pecari*	W. L. peccary	0	0	0	0	0	0

*n.i.*: not identified.

### Comparisons between mature forest (MF) and mosaic (MC)

Six of the 11 species recorded (the lowland tapir, collared peccary, raccoon, paca, agouti and fox) had only low occurrence over the 10 blocks sampled in the landscape (i.e., were found in two to four blocks). Thus, we had too few sampling replicates in the landscape (i.e., blocks with fauna records) to address statistically and suitably the use of MFs and MCs by these species.

Conversely, the recordings for deer (recorded in seven blocks), armadillo (in nine blocks), and opossum, tayra and coati (all recorded in the 10 blocks) in most of the sampling replicates made this analysis suitable. For this reason, we considered only these five species in the statistical analyses concerning frequency of use of the MFs and MCs.

**Figure 3:**
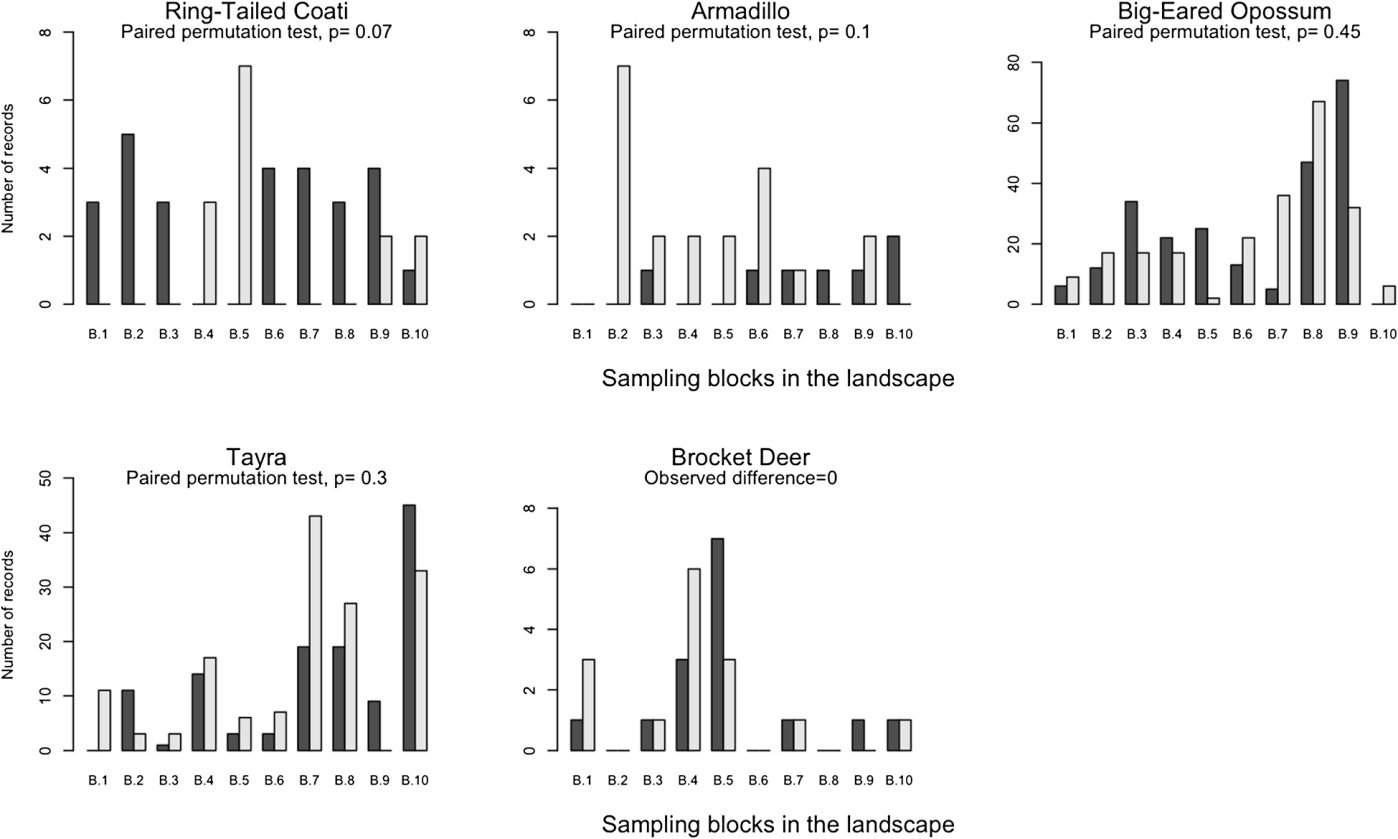
**Number of species recordings in the MF (dark bars) and MC (light bars) contexts.** Data from each of the 10 blocks sampled in the landscape (*quilombola* areas in the Ribeira Valley).

The opossum, tayra, armadillos and deer showed a non-selective pattern in the use of these two environments. In contrast, the coati was consistently more common in MF (Figure 3). Regarding LEK, we found that, on average, 38% of the responses of the interviewees converged with the scientific data on the use of MF and MC considering the five species statistically analyzed.

A total of 42% of the responses of the interviewees converged with the scientific data reporting that the tayra uses MFs and MCs with the same frequency. Among those who diverged from the scientific data, most (36%) of the respondents indicated a preference for the MF environment, and only 21% indicated the MCs as the environment preferred by the species. Regarding the coati, only 27% of the interviewees’ responses converged with the scientific data, reporting greater species’ use of MFs. Another 27% believed in a higher coati occurrence in MCs, while most of the interviewees (45%) reported that there was no species habitat selection.

In the case of the opossum, 32% of the interviewees converged with the scientific data, stating no species’ preference for MFs or MCs. However, most (56%) of the respondents argued that the opossum is most easily found in MCs, and only 12% of them reported that this species uses MFs the most. Regarding the armadillo, 44% of the respondents suggested there is no species’ selection of the environment, matching the faunal record. In addition, 56% of the interviewees stated that armadillos use MCs the most. Thus, there was complete agreement among the interviewees on the armadillos’ non-preferential use of MFs.

Regarding deer, 44% of the interviewees converged with the scientific data on the species’ non-selection of habitats. A total of 42% of the interviewees said that this species uses MCs the most, and 15% believed that the deer preferred MFs.

### Comparisons among the types of *capoeiras*

The coati and deer were recorded only in four of the 10 MCs sampled in the landscape. With a pattern of such low occurrence (so few sampling replicates), we did not include these species in our statistical analyses regarding the use of *capoeiras* by fauna. Thus, we only statistically analyzed the cases of the armadillo, tayra and opossum, which were recorded in seven, nine, and 10 MCs, respectively.

According to the camera traps, no preferential pattern of use for a particular type of *capoeiras* was detected for these mammals (P = 0.7, 0.3 and 0.8 for the armadillo, tayra and opossum, respectively). Regarding LEK, 50% of the interviewees converged, on average, with the scientific data, reporting that the tayra, armadillo and deer, when found in the mosaic areas, use the three types of *capoeiras* with no pattern of preference. LEK divergences relative to scientific data were not related to any particular type of *capoeira*.

### Species life habits (diurnal/nocturnal)

The camera recordings showed a clear pattern of mostly diurnal species, including the coati, tayra and agouti, and those of nocturnal habits, including the opossum, armadillo, crab-eating fox, deer, paca and raccoon. The lowland tapir and collared peccary, with only five and three recordings, respectively, were not considered in this analysis (Table 2). We found high levels of internal agreement and LEK convergence with the scientific data regarding this aspect of species biology, except for the knowledge concerning the tayra and the agouti (Table 2).

**Table 2:** Species’ activity periods, according to the percentage of photographic recordings (P) and the percentage of interviewees (I)

**Species**	**Daytime (P)**	**Nighttime (P)**	**Diurnal (I)**	**Nocturnal (I)**	**Cathemeral (I)**
Ring-tailed coati	78	22	82	9	9
Opossum	1	99	0	100	0
Armadillo	0	100	6	86	8
Tayra	93	7	48	27	24
Azara’s agouti	91	9	45	39	15
Crab-eating fox	11	89	13	66	22
Brocket deer	7	93	9	82	9
Lowland paca	0	100	0	100	0
Raccoon	0	100	6	85	9
Lowland tapir	*n.d.*	*n.d.*	19	69	13
W. L. peccary	*n.d.*	*n.d.*	67	6	27

*n.d.*: There was no data on the species’ life habit.

The data were based on 36 interviewed *quilombolas* in the Ribeira Valley.

### Internal agreement and convergence patterns on species’ habitat use

**Figure 4:**
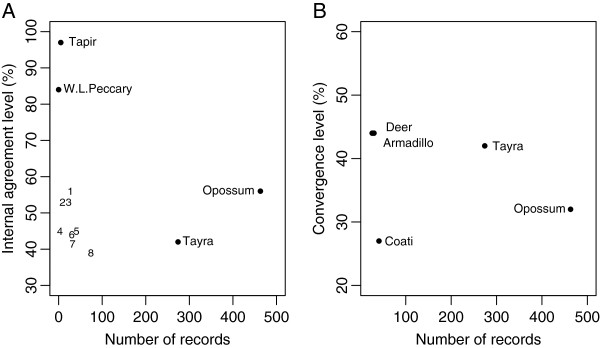
**Correlation patterns between ethnoecological and ecological data. (A)** Association between internal agreement levels and the number of species recordings. **(B)** Association between convergence levels and the number of species recordings. The data were based on knowledge of 36 *quilombolas* about species’ habitat use in the Ribeira Valley (1: Armadillo; 2: Fox; 3: Raccoon; 4: C. Peccary; 5: Coati; 6: Deer; 7: Agouti; 8: Paca).

We noticed a trend toward a higher degree of internal agreement involving the rarest mammals in the area, including the lowland tapir and white–lipped peccary (Figure 4A). The tayra is another example of this negative correlation. The tayra is the third most common species on the site and one of those with the lowest level of agreement among the interviewees regarding the species’ habitat use (Figure 4A).

Only five mammals were recorded in sufficient numbers to analyze the convergence between the LEK and the scientific data on species’ habitat use. No correlation was found between the degree of convergence and the number of records for each species in the area (Figure 4B).

## Discussion

### Use of secondary forests by species

There has been a broad discussion in the ecological literature regarding the prevalence of secondary forests and their conservation potential in the tropics during the last decade [30,53-57]. Studies have also diverged regarding the specific effects of the shifting cultivation system on biodiversity. This process is either associated with the maintenance of or an increase in biodiversity in general [31,58-61], or it is considered to be one of the main causes of its decrease in the environments thereby affected [62-64].

In the present study, we showed that the tayra, armadillo, opossum and deer use secondary forests (resulting from shifting cultivation activities) and mature forests with the same frequency, while the coati prefers mature forests. Thus, the old plantations undergoing a forest-regeneration process were as attractive as adjacent mature forests to four of the five species analyzed.

It is noteworthy that in the context of *quilombola* communities in Ribeira Valley, the field areas traditionally created by shifting cultivation ranged from 0.5 to 1 ha and were included in a matrix of native forests (old plantation fields at various stages of regeneration) [26,27]. Shifting cultivation locally promoted high landscape heterogeneity through its fallow-in-rotation system, generating mosaics formed by patches of secondary vegetation with different ages.

Furthermore, this agricultural practice did not historically result in local forest fragmentation [25,26,28], a process that has been threatening the Atlantic Forest Biome in almost all of its area [65] and the associated large mammals [66-68].

Additionally, the results shown herein converge with those of studies regarding the effects of shifting cultivation practices on large mammals in Zaire [69] and Peru [70], small mammals in Mexico [31], primates in Africa [71,72] and birds in Colombia [58] and Guatemala [73]. For a more comprehensive review of the subject, refer to [30].

### Levels of internal agreement and convergence with the scientific data

First, it is worth highlighting the high levels of convergence regarding the species’ life habits (diurnal/nocturnal), except for the tayra and agouti cases. However, we found a pattern of lower convergence in the knowledge about the use of the environments by the mammals.

The lack of a positive correlation between the number of species recorded and the variables “degree of internal agreement” and “convergence level” (Figure 4A and B) suggests that more frequent species occurrence in the area is not directly related to more consensual knowledge among the people or even to a notion closer to the animal’s current pattern of habitat use (that is, higher convergence).

For example, the level of internal agreement (42%) on the tayra’s habitat use was the second lowest (Figure 4A), although the tayra was the second-most frequently recorded species in the area (274 recordings) (Table 1). Moreover, the tayra is among the three species (along with the agouti and crab-eating fox) for which two sets of knowledge on that animal’s diurnal or nocturnal life habits disagreed (Table 2).

Thus, the low levels of internal agreement and convergence suggest that the tayra is not well known by the population studied herein despite it being among the most common mammals on the site (Table 1), predominantly showing diurnal habits (Table 2) and occurring in the MC context (more closely associated with the people’s activities in the landscape) as much as in MFs (paired permutation test, P = 0.3).

Reports indicating extremely rare encounters between people and tayras were also common during the interviews. Thus, we suggest here that the species’ elusive behavior in this area is likely the main cause of the low level of related knowledge by the people.

Unlike the tayra, the lowland tapir stood out for its related high levels of internal agreement in the LEK, mainly because the lowland tapir is extremely rare. A total of 97% of the interviewees noted the higher frequency of lowland tapirs in the MF contexts.

Furthermore, reports of the alleged remoteness of lowland tapirs, which were claimed to take refuge in the region’s main nature reserves, were common. The residents’ perception also aligns with data from a faunal survey performed at Intervales State Park (bordering the communities studied herein). Lowland tapirs were recorded at much higher rates in the area within the park than in the communities (unpublished data). Accordingly, we hypothesized that the interviewees were referring to the current near-absence of lowland tapirs in the communities as a whole when they responded that this animal is almost always found in MFs.

The white-lipped peccary case (with an internal agreement level of 84% in the population) seems to be similar to that of the lowland tapir, and this peccary is extremely rare throughout its range in the Neotropics [74-76]. The large groups of this animal, formed by hundreds of individuals, forage extensive areas [77], which makes them highly vulnerable to large-scale anthropic activities [78], including extensive agriculture and animal husbandry. Finally, in an exploratory approach, we found that the age of the interviewees (within the 40+ population) had no influence on the internal agreement and convergence variables.

### The relevance of the mosaic context in the LEK

In this study, the hypothesis that MCs would have a greater relevance for local knowledge of the use of space by these species was validated for raccoon, opossum, deer and armadillo and rebutted only for tayra. This result enabled the identification of the landscape portion (MCs) with the highest relevance for the development of the *quilombola* knowledge regarding the use of the landscape by the mammals analyzed.

Theoretically, we based the interpretation of our results on the premise that human perception of the environment—and the resulting ecological knowledge—is best understood as the result of a core set of people’s experiences and practices in the environment [79-81]. Therefore, we argue that the evidence for a greater emphasis of this *quilombola* LEK on the MCs suggests that this environment must have been the main context of the people’s experience in the landscape and the most significant environment during the development of this knowledge.

This line of reasoning converges with previous studies indicating that the areas related to the communities’ main subsistence activities - such as crop areas, fallows, houses, gardens and areas for animal husbandry - was concentrated in the MCs context until the early 1980s [25-28].

## Conclusions

Based on the results reported herein, we introduce some considerations of both ecological and ethnoecological interest: (1) *in situ* recording of large-bodied mammals still represents a challenge for ecologists and zoologists given the difficulties in recording that fauna group; (2) for four of the five species analyzed, the anthropogenic context of the landscape—formed by *capoeiras* at different stages of re-growth—was as attractive as the mature forest environment; (3) the local knowledge of the species’ life habits (diurnal/nocturnal) was consensual and highly convergent with the scientific data, while that involving habitat use showed higher divergence among the interviewees.

The low level of internal agreement on the species’ environment use showed, similarly to other studies [10,82-84], how LEK might vary within a given population. Given these idiosyncrasies, it is worth emphasizing the significance of using an ethnographic approach prior to LEK surveys in studies focused on the complementarity between science and local knowledge. A more detailed knowledge of peoples’ experiences in the environment can guide a more heterogeneous selection of interviewees, thus enhancing the recording of complementary aspects of LEK regarding the corresponding scientific knowledge [11,85].

The identification of singularities of local and scientific knowledge systems has also been a topic of interest in studies of LEK [85,86]. On that subject, we highlight herein a LEK special focus targeted toward the anthropogenic portion of the landscape. Indeed, this type of environmental focus in LEK has not previously been considered as part of what makes LEK different from scientific knowledge and unique.

## Abbreviations

LEK: Local ecological knowledge; MC: Mosaic of *capoeiras*; MF: Continuum of mature forest; C1: Initial regeneration stage *capoeira*; C2: Medium regeneration stage *capoeira*; C3: Advanced regeneration stage *capoeira*.

## Competing interests

The authors declare that they have no competing interests.

## Authors’ contributions

HMP conceived and designed the study, carried out the field survey and analyses, interpreted the results and wrote the manuscript. RSSM supervised the research, made substantial contributions to the theoretical background, helped in the interpretation of the results and participated in writing the manuscript. CA contributed to theoretical background as well as to fieldwork. She participated in the interpretation of the results and helped write the manuscript. ESB contributed to developing the theoretical background and provided valuable insights for data analysis and the interpretation of the results. He also contributed to the writing of the manuscript. All authors approved the final manuscript.

## Authors’ information

HMP is a biologist and currently carrying out post-doctoral work at the University of São Paulo, Brazil. Main research interest: Human Ecology and Ethnoecology among Amazonian and Atlantic Forest populations. RSSM is a Professor of Anthropology at the University of São Paulo, Brazil. He coordinates the Human Ecology Division of the Laboratory for Human Evolutionary Studies. Main research field: nutritional and environmental anthropology of Amazonian and Atlantic Forest people. CA is a Professor at the School of Arts, Sciences and Humanities at the University of São Paulo, Brazil. She coordinates the Laboratory of Human Ecology and is a member of the Research Center on Complex System Modeling. Main research interest: adaptability of peasant populations to the neotropical rainforests (Amazon and Atlantic Rainforest). ESB is a Professor of Anthropology at the Indiana University, Bloomington, US. Main research field: historical analysis of land use change, people-forest interaction, household studies and ethnobotany among Amazonian caboclo and colonist populations.
